# Role of Chronic Administration of Antidepressant Drugs in the Prenatal Stress-Evoked Inflammatory Response in the Brain of Adult Offspring Rats: Involvement of the NLRP3 Inflammasome-Related Pathway

**DOI:** 10.1007/s12035-018-1458-1

**Published:** 2019-01-04

**Authors:** Ewa Trojan, Katarzyna Chamera, Natalia Bryniarska, Katarzyna Kotarska, Monika Leśkiewicz, Magdalena Regulska, Agnieszka Basta-Kaim

**Affiliations:** Department of Experimental Neuroendocrinology, Institute of Pharmacology, Polish Academy of Sciences, 12 Smętna St., 31-343 Kraków, Poland

**Keywords:** Antidepressant drugs, Prenatal stress, NLRP3 inflammasome, Proinflammatory factors

## Abstract

Evidence indicates that adverse experiences in early life may be a factor for immune disturbances leading to the depression in adulthood. Recently, a pivotal role in the pathogenesis of depression has been assigned to the activation of the brain Nod-like receptor pyrin-containing 3 (NLRP3) inflammasome. We investigated the impact of chronic treatment with antidepressant drugs on the behavioral disturbances and the levels of proinflammatory factors in the hippocampus and frontal cortex of adult male rats after prenatal stress exposure. Next, we explored the involvement of the NLRP3 inflammasome-related pathways in the mechanism of antidepressant action. Our study confirmed that chronic antidepressant treatment attenuated depression-like disturbances and exerted an anxiolytic action. All antidepressants diminished the prenatal stress-induced increase in IL-1β in both brain areas, while IL-18 only in the hippocampus. Moreover, tianeptine administration diminished the increase in CCR2 levels in both brain areas, while in the hippocampus, tianeptine, along with venlafaxine CCL2 and iNOS levels. Next, we observed that in the hippocampus, tianeptine and fluoxetine suppressed upregulation of TLR4. Furthermore, venlafaxine suppressed NFкB p65-subunit phosphorylation, while fluoxetine enhanced the IкB level. Importantly, in the hippocampus, all antidepressants normalized evoked by stress changes in caspase-1 level, while tianeptine and venlafaxine also affect the levels of ASC and NLRP3 subunits. Our results provide new evidence that chronic administration of antidepressants exerts anti-inflammatory effects more pronounced in the hippocampus, through suppression of the NLRP3 inflammasome activation. These effects are accompanied by an improvement in the behavioral dysfunctions evoked by prenatal stress.

**Electronic supplementary material:**

The online version of this article (10.1007/s12035-018-1458-1) contains supplementary material, which is available to authorized users.

## Introduction

A vast body of evidence suggests that depression is a complex disorder involving molecular, structural, and functional dysfunctions in several brain areas, which makes the biological background of this illness still unclear [1]. Due to the complexity of depression, commonly used pharmacological therapeutic schemes are only effective in approximately 50% of patients, and many patients respond to these medications only after a long-lasting treatment period, which often leads to side effects [2]. Therefore, there is still a need to conduct studies on the background of depression and to identify new intracellular targets for antidepressant drug action, which may help stratify patients and deliver tailored treatments.

Among a number of hypotheses of depression, the immune theory postulates that functional changes in the immune system and its mediator cytokines and chemokines may be crucial in the development of this disease [3–5]. During neuroinflammation, harmful mediators, such as nitric oxide (NO) and reactive oxygen species (ROS), can participate in stress-induced depression [6]. Chemokine CC ligand 2 (CCL2) and its receptor CC receptor 2 (CCR2) are important modulators of chemotaxis of monocyte-derived macrophages and other inflammatory cells to the disturbed brain area [7, 8]. CCL2 expressed in the brain, mostly in the hippocampus and cortex, is also implicated in neuronal communication and neuroendocrine regulation, while its colocalization with classical neurotransmitters, such as acetylcholine, dopamine, and GABA, indicates a wider role of the CCL2–CCR2 axis in the brain [9]. Interestingly, the pleiotropic actions of this chemokine are likely to be relevant not only to the pathophysiology of psychiatric disorders in adulthood [10] but also potentially to the developmental pathogenesis of depression, as suggested by its extensive and dynamic expression during in utero neurodevelopment.

In the context of our study, the regulatory role of CCL2 in microglia under basal and inflammatory conditions is crucial [7, 11]. Data have demonstrated that the CCL2–CCR2 axis may enhance and prolong microglia activation, release proinflammatory factors, such as interleukin-1β (IL-1β) and IL-18, activate iNOS [12], and through a feedback mechanism upregulate both CCL2 and CCR2 in response to the mentioned cytokines [11]. Many studies have shown that IL-1β and IL-18 are involved in diverse signs of immune response and the initiation, regulation, and maintenance of inflammation as well as in the modulation of neuroimmune pathways that regulate brain circuits relevant to reward, mood, and cognition [13, 14]. Furthermore, several reports have associated changes in IL-1β and IL-18 levels and signaling with depressive symptoms. For example, an epidemiological study found enhanced levels of IL-1β in the peripheral circulation and cerebral spinal fluid (CSF) of depressed patients [15, 16]. Preclinical studies have shown that IL-1β administration modified behavioral and neurochemical processes considered relevant to mood regulation, e.g., in rats, intracerebroventricular treatment with IL-1β and enhanced serum IL-1β levels were associated with depressive-like and anhedonia behavior [17]. Along these lines, chronic treatment with IL-1Ra diminished the malfunction of microglia migration and the depressive-like behavior observed in a chronic unpredictable stress animal model of depression [18]. Similarly, the role of IL-18 in homeostasis and behavior modulation is commonly accepted [19]. In addition, clinical data revealed increased peripheral (plasma) levels of IL-18 in patients with depression [20, 21], while an experimental study described elevated neocortical IL-18 gene expression in animal model of stress and depressive behavior based on social defeat [22].

Recently, many findings have indicated that IL-1β and IL-18 are the main cytokines controlled by the Nod-like receptor pyrin-containing 3 (NLRP3) inflammasome activation [23]. NLRP3, a multiprotein complex consisting of NLRP3, pro-caspase-1, and apoptosis-associated speck-like protein containing a caspase recruitment domain (ASC), is highly expressed in microglia and important in the development of the neuroinflammation [24]. Experimental data indicated that the activation of NLRP3 was regulated both at transcriptional and posttranslational levels. The first signal in inflammasome activation involves “priming” induced by the toll-like receptor (TLR) and nuclear factor (NFкB) pathways to upregulate transcription of proIL-1β, proIL-18, and the NLPR3 inflammasome, the level of which under basal conditions is relatively low, and followed by its translocation to the cytoplasm [25]. The second stimulus activates the NLRP3 inflammasome by facilitating the oligomerization of inactive NLRP3, ASC, and procaspase-1. This complex in turn catalyzes the conversion of procaspase-1 to caspase-1, contributing to the production and secretion of mature cytokines, mainly IL-1β and IL-18. Recently, inflammasomes captured scientific interest as accurate sensors of brain homeostasis malfunction in the course of stress-related disorders. In fact, acute immobilization stress led to NLRP3 activation in the hippocampus [26]. Moreover, the lack of susceptibility of NLRP3-null mice to depressive behaviors, including anhedonia induced by chronic stress, and limited IL-1β release in the brain was demonstrated [27]. Interestingly, clinical data indicated that peripheral blood mononuclear cells (PBMCs) of depressed patients showed not only elevated IL-1β and IL-18 levels but also activated NLRP3 inflammasome [28].

An important question that arises is whether drugs currently used in the pharmacotherapy of depression exhibit anti-inflammatory potential, particularly in brain immune cells, via an impact on NLRP3 inflammasome-related pathways. So far, data concerning this subject are limited. Therefore, the present study was designed to explore the impact of chronic treatment with antidepressant drugs with various mechanisms of action, i.e., tianeptine (an atypical antidepressant, which was found to selectively potentiate serotonin uptake into rat brain synaptosomes [29]), venlafaxine (a serotonin and norepinephrine reuptake inhibitor), and fluoxetine (a serotonin reuptake inhibitor), on the behavioral changes evoked by a prenatal stress procedure (regarded as an animal model of depression). In the set of biochemical experiments, we evaluated the impact of tianeptine, venlafaxine, and fluoxetine on the protein expression of the proinflammatory cytokines IL-1β, IL-18, chemokine CCL2, and chemokine CCL2 receptor (CCR2) as well as iNOS levels in the hippocampus and frontal cortex of prenatally stressed male offspring. Throughout all experiments, to study the putative mechanisms underlying the potentially beneficial effects of chronic antidepressant treatment, we focused on the intracellular NLRP3 inflammasome signaling pathways (e.g., TLR4/MyD88 and NFкB) related to its activation and in consequence to the production of proinflammatory factors in the brain.

## Materials and Methods

### Animals

Sprague–Dawley rats (Charles River, Sulzfeld, Germany) were maintained under standard conditions (at room temperature of 23 °C, 12/12 h light/dark cycle), with food and water available ad libitum. To determine the estrous cycle phase, vaginal smears were obtained daily from the female rats. On the proestrus day, the females were placed with males for 12 h and the next morning, they were checked for the presence of sperm in the vaginal smears. Pregnant females were randomly assigned to control and stress groups (*n* = 10 in each group). All experimental protocols were approved by the Local Ethics Committee in Kraków, Poland (approval no. 1037/2013, 16 May 2013).

### Stress Procedure

**Fig. 1:**
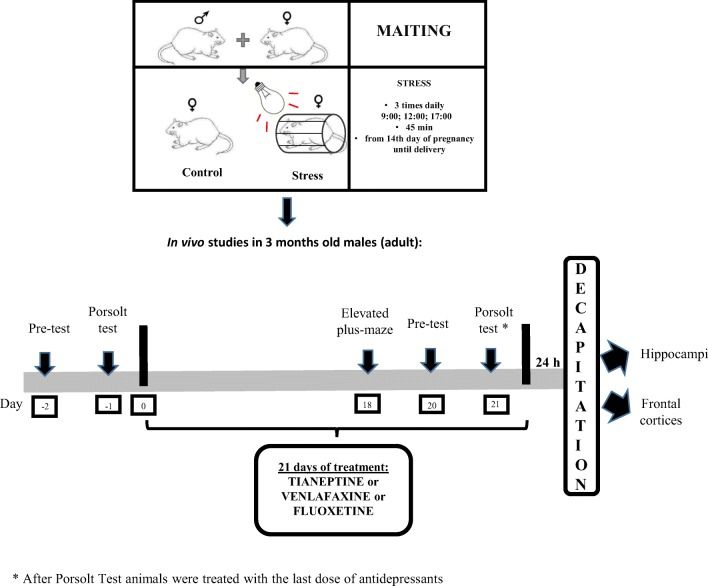
Schedule of the experiment

The prenatal stress procedure was conducted as previously described [30–33]. Briefly, pregnant females were subjected to stress sessions daily (at 9:00 am, 12:00 pm and 5:00 pm), beginning on the 14th day of pregnancy until delivery. In each session, rats were placed in plastic cylinders (7 × 12 cm) and exposed to bright light (150 W) for 45 min. Control pregnant females were left undisturbed in their home cages. For all experiments, male offspring were selected from 21-day-old litters. They were housed in groups of five animals per cage (one or two animals from each litter) under standard conditions. At 3 months of age, the offspring of the control and stressed mothers underwent the first behavioral verification in the forced swim test (Fig. 1).

### Forced Swim Test (FST, Porsolt Test)

The FST was performed according to the method described by Detke et al. [34]. Animals were subjected to two trials (individually) during which they were forced to swim in a cylinder (50 cm high, 18 cm in diameter) filled with water (23 °C) to a height of 35 cm. Twenty-four hours after the first trial (pretest), the second trial (test) was conducted. The first trial lasted 15 min, while the second trial lasted 5 min. As previously described, the total durations of immobility, mobility (swimming), and climbing were measured throughout the second trial [31, 32, 34–36].

### Antidepressant Drug Administration

After the FST (behavioral verification), the control and prenatally stressed male rats were divided into eight experimental groups (CONTROL+VEH, CONTROL+FLU, CONTROL+VEN, CONTROL+TIA, STRESS+VEH, STRESS+FLU, STRESS+VEN, STRESS+TIA; six animals per group). They were injected intraperitoneally once daily with antidepressant drugs: fluoxetine (Eli Lilly, Indianapolis, IN, USA), venlafaxine (Sequoia Research, Pangbourne, UK), and tianeptine (Tocris Bioscience, Bristol, UK) at a dose of 10 mg/kg. All drugs were dissolved in 0.9% saline. The controls were treated with 0.9% saline (Polpharma, Starogard Gdański, Poland).

On the last days of chronic treatment with antidepressants, animals underwent the elevated plus-maze test and the forced swim procedure again (for pharmacological verification of the animal model of depression, according to the schedule illustrated in Fig. 1).

### Elevated Plus-Maze Test

The elevated plus-maze test was performed as previously described by Pellow et al. [37]. The maze was elevated to a height of 50 cm above the floor and illuminated from below by a dim light (15 W). To allow the animals to habituate to the conditions in the experimental room, they were placed there for 1 h before the test. Each rat was individually placed in the junction of the open and closed arms, facing a closed arm, and observed for 5 min. An entry was recorded when the animal entered the arm with all four limbs. The behavioral study was not blinded.

### Tissue Collection

Rats were sacrificed by rapid decapitation 24 h after the last injection of antidepressant drugs. Brain structures, i.e., the frontal cortices (FCx) and hippocampi (Hp), from all rats were dissected, and the tissues were immediately frozen on dry ice and stored at − 80 °C.

### Tissue Preparation and Determination of Protein Concentration

All tissue samples were homogenized in 2-ml Eppendorf ® tubes filled with an appropriate buffer using a Tissue Lyser II (Qiagen Inc., Valencia, CA, USA). All sample extracts were diluted and stored at − 20 to − 80 °C until use. In all experiments, the protein content analyses of all the samples were performed using a BCA Protein Assay Kit (Sigma Aldrich, St. Louis, MO, USA) according to the supplier’s instructions, and the protein contents were measured using a Tecan Infinite 200 Pro spectrophotometer (Tecan, Männedorf, Germany). Samples prepared in this way were used for ELISA, and Western blot analysis. A portion of the samples was used for the determinations described previously in Trojan et al. [38].

### Enzyme-Linked Immunosorbent Assay (ELISA)

For each ELISA test, the samples were prepared in accordance with the supplier’s recommendations.

The levels of CCL2 (ELISA kit for monocyte chemotactic protein 1 (CCL2/MCP-1); USCN Life Science Inc., Wuhan, China), CCR2 (Rat Chemokine Receptor Type 2 (CCR2) ELISA kit; Cusabio, Houston, TX, USA), IL-1β, IL-18, IL-4 (ELISA kit for Rat Interleukin 1β (IL-1β), ELISA kit for Rat Interleukin 18 (IL-18); all from USCN Life Science Inc., Wuhan, China), NLRP3, ASC (ELISA kit for Pyrin Domain-Containing Protein 3, ELISA kit for PYD and CARD Domain-Containing Protein; both from USCN Life Science Inc., Wuhan, China), Casp-1 (caspase-1 ELISA kit; EIAab Wuhan Science, Wuhan, China), and iNOS (Rat Inducible Nitric Oxide Synthase ELISA kit; Cusabio, Houston, TX, USA) in the cortical and hippocampal homogenates were measured using a commercially available ELISA kits. The detection limits were as follows: CCL2, 0.064 ng/mL; CCR2, 3.9 pg/mL; IL-1β, 2.64 pg/mL; IL-18, 5.9 pg/mL; NLRP3, 0.123 ng/mL; ASC, 0.065 ng/mL; Casp-1, 78 pg/mL; and iNOS, 0.195 IU/mL. Interassay precision was as follows: CCL2, < 12%; CCR2, < 10%; IL-1β, < 12%; IL-18, < 12%; NLRP3, < 12%; ASC, < 12%; Casp-1, < 7.8%; and iNOS, < 10%. Intra-assay precision was as follows: CCL2, < 10%; CCR2, < 8%; IL-1β, < 12%; IL-18, < 12%; IL-4, < 12%; NLRP3, < 10%; ASC, < 10%; Casp-1, < 5.3%; and iNOS, < 8%. Positive controls for each assay were provided by the manufacturers.

### Western Blot

Samples containing equal amounts of protein were mixed with 4× Laemmli sample buffer (Bio-Rad, Hercules, CA, USA) and heated at 95 °C for 5 min. Proteins were separated using 4–20% Criterion™ TGX™ Precast Midi Protein Gel, 26 well (Bio-Rad, Hercules, CA, USA) under constant voltage (200 V) and then transferred electrophoretically to PVDF membranes (Trans-Blot Turbo; Bio-Rad, Hercules, CA, USA). Next, the membranes were incubated overnight at 4 °C with the appropriate primary antibodies: anti-phospho-p-65 (sc-33039), anti-IκB (sc-1643), anti-phospho-p38 (sc-101759), anti-phospho-ERK1/2 (sc-16982), anti-phospho-JNK (sc-12882), anti-phospho-PI3K (sc-1637), and anti-TLR4 (sc-293072) (all antibodies were from Santa Cruz Biotechnology, Inc., Dallas, TX, USA). All antibodies had been diluted in a SignalBoost Immunoreaction Enhancer Kit (Millipore, Warsaw, Poland). The next day, after washing four times, membranes were incubated with a peroxidase-labeled secondary antibody (anti-rabbit/anti-mouse IgG; Vector Laboratories, Burlingame, CA, USA) at room temperature for 1 h. After the incubation, the membranes were rinsed with a large volume of TBST (Tris-buffered saline (TBS, pH = 7.5) containing 0.1% Tween-20). The immune complexes were detected using the Pierce® ECL Western Blotting Substrate (Thermo Fisher, Pierce Biotechnology, Carlsbad, CA, USA) and visualized using a Fujifilm LAS-1000 System (Fuji Film, Tokyo, Japan). After phospho-MAPK, phospho-PI3K, and IкB determination, the blots were stripped in stripping buffer containing 100 μL of Tris–HCl (pH = 6.7), 2% SDS, and 700 μL of 2-mercaptoethanol (all from Sigma Aldrich, St. Louis, MO, USA) and reprobed with antibodies against unphosphorylated MAPK: anti-p-65 (sc-372), anti-p-38 (sc-7149), anti-ERK1/2 (sc-135900), anti-JNK (sc-7345), anti-PI3K (sc-12929), and against Myd88 (ab2064; Abcam, Cambridge, UK). After a second stripping, membranes were stripped again and reprobed with an antibody against β-actin (MAB374; Millipore, Warsaw, Poland) diluted in SignalBoost Immunoreaction Enhancer Kit for normalization of all bands. The relative levels of immunoreactivity were densitometrically quantified using Fujifilm Multi Gauge software (Fuji Film, Tokyo, Japan).

### Statistical Analysis

All of the statistical analyses were performed using Statistica software, version 10.0 (Statsoft, Tulsa, USA). The outcomes of the behavioral studies are presented as the mean ± SEM. The data obtained in the ELISA study are presented as weight units (pg or ng) per milligram of protein ± SEM; and for Western blot analysis, the results are presented as the percentage of the control ± SEM. The normality of variable distribution and homogeneity of variances were checked by the Shapiro–Wilk test and Levene’s test, respectively. The significance of the differences between the means was evaluated by one- or two-way analysis of variance (ANOVA), with Duncan’s post hoc test if appropriate. A value of *p* < 0.05 was considered statistically significant. All data are presented as the mean ± SEM (standard error of the mean). All graphs were prepared using GraphPad Prism 7.

## Results

### Behavioral Study

#### Chronic Administration of Antidepressant Drugs Attenuated Changes, Evoked by Maternal Stress, in Behavioral Parameters in Adult Offspring Rats

##### Forced Swim Test

As we showed previously [38], rats after prenatal stress displayed depressive-like behavior. In fact, the prenatal stress procedure significantly prolonged immobility time in the forced swim test (*F*_1,57_ = 100.65; *p* < 0.05; 192.5 ± 26.9 Control (Con) vs. 252.55 ± 18.02 Stress (PS)) and lowered swimming (*F*_1,57_ = 100.66; *p* < 0.05; 107.5 ± 26.9 Con vs. 47.44 ± 18.02 PS) and climbing times (*F*_1,57_ = 54,56; *p* < 0.05; 92.13 ± 30.63 Con vs. 43.44 ± 18.22 PS) (Table 1).

**Table 1:** The effect of prenatal stress on the times for immobility, swimming, and climbing in the forced swim test

Forced swim test		
	Control	Prenatal stress
Immobility (s)	192.5 ± 26.9	252.55 ± 18.02*
Swimming (s)	107.5 ± 26.9	47.44 ± 18.02*
Climbing (s)	92.13 ± 30.63	43.44 ± 18.22*

The results are presented as the mean ± SEM. Statistics: one-way ANOVA

*p < 0.05 in comparison to control group, *n* = 24–26 for each group

Next, to determine whether chronic tianeptine, venlafaxine, or fluoxetine administration affected the behavioral changes evoked by prenatal stress, we performed the FST in rats again. As we previously demonstrated [38], enhanced immobility time (*p* < 0.05; 201 ± 4.8 Con vs. 275.16 ± 1.4 PS) and shortened swimming (*p* < 0.05; 99 ± 4.8 Con vs. 24.83 ± 1.4 PS) and climbing (*p* < 0.05; 51.5 ± 3.37 Con vs. 16.16 ± 0.74 PS) times were detected in prenatally stressed offspring compared with control offspring, which led to the conclusion that the behavioral disturbances evoked by prenatal stress are long lasting (Table 2). We also confirmed a significant effect of drugs (*F*_3,39_ = 13.02; *p* < 0.05) on the immobility time. Post hoc comparisons revealed an effect of tianeptine (*p* < 0.05; 275.16 ± 1.4 PS vs. 229.56 ± 2.93 PS + Tia), venlafaxine (*p* < 0.05; 275.16 ± 1.4 PS vs. 229.56 ± 8.27 PS + Ven), and fluoxetine (*p* < 0.05; 275.16 ± 1.4 PS vs. 248.8 ± 1.28 PS + Flu). Furthermore, a significant effect of chronic administration of the drugs (*F*_3,39_ = 13.02; *p* < 0.05; Table 2) on swimming time was observed. Post hoc comparisons revealed that tianeptine (*p* < 0.05; 24.83 ± 1.4 PS vs. 70.33 ± 0.04 PS + Tia), venlafaxine (*p* < 0.05; 24.83 ± 1.4 PS vs. 70.33 ± 8.27 PS + Ven), and fluoxetine (*p* < 0.05; 24.83 ± 1.4 PS vs. 51.2 ± 1.28 PS + Flu) extended the swimming time in stressed offspring compared to control offspring. Regarding climbing time, we observed that only tianeptine (*F*_3,38_ = 7.94; *p* < 0.05; 16.16 ± 0.74 PS vs. 35.66 ± 0.88 PS + Tia) prolonged the climbing in prenatally stressed rats compared to control rats (Table 2; [38]).

**Table 2:** The effects of prenatal stress (PS) and chronic antidepressant drugs treatment (tianeptine (Tia), venlafaxine (Ven), or fluoxetine (Flu)) on the immobility, mobility, and climbing time (in seconds) in the forced swim test and the number of visits and the time spent in the open arms of the elevated plus-maze

	Control	PS	Control + Tia	PS + Tia	Control + Ven	PS + Ven	Control + Flu	PS + Flu
Forced swim test (FST, Porsolt test)								
Immobility (s)	201 ± 4.8	275.16 ± 1.4*	181.33 ± 8.02	229.56 ± 2.93#	221.5 ± 8.32	229.56 ± 8.27#	218 ± 7.22	248.8 ± 1.28#
Swimming (s)	99 ± 4.8	24.83 ± 1.4*	118.66 ± 8.02	70.33 ± 0.04#	78.5 ± 8.32	70.33 ± 8.27#	82 ± 7.22	51.2 ± 1.28#
Climbing (s)	51.5 ± 3.37	16.16 ± 0.74*	49.66 ± 4.72	35.66 ± 0.88#	73.3 ± 8.47	50.66 ± 11.24	64.6 ± 3.33	25 ± 3.11
Elevated plus-maze								
Number of visit in the open arms	2.00 ± 0.44	0.33 ± 0.23*	1.75 ± 0.42	3.2 ± 0.86#	2.20 ± 0.58	3.66 ± 0.73#	0.80 ± 0.49	0.40 ± 0.6
Time spent in the open arm (s)	17.00 ± 3.88	1.33 ± 1.46*	10.75 ± 1.23	49.25 ± 9.72#	20.16 ± 9.69	49.80 ± 9.54#	12.6 ± 7.75	13.20 ± 3.97

The data are presented as the means ± SEMs, with *n* = 5–6 for each group

∗*p* ≤ 0.05 vs. control Veh group; ^#^*p* ≤ 0.05 vs. prenatally stressed Veh group. ANOVA (two-way), followed by Duncan’s test

##### Elevated Plus-Maze Test

The elevated plus-maze test was performed to assess anxiety-like behavior in adult rats. Similar to our previous reports [38, 39], we confirmed that, compared to control rats, those exposed to the prenatal stress procedure had a significant reduction in the number of entries into the open arms (*F*_1,34_ = 32.46; *p* < 0.05; 2.00 ± 0.44 Con vs. 0.33 ± 0.23 PS; Table 2) and a significant decrease in the time spent in the open arms of the maze (*F*_1,34_ = 88.57; *p* < 0.05; 17.00 ± 3.88 Con vs. 1.33 ± 1.46 PS; Table 2). In line with our previous observations, post hoc comparisons showed that tianeptine (*p* < 0.05) and venlafaxine (*p* < 0.05) significantly enhanced, relative to vehicle, the number of entries into the open arms of the maze (*p* < 0.05; 0.33 ± 0.23 PS vs. 3.2 ± 0.86 PS + Tia; 0.33 ± 0.23 PS vs. 3.66 ± 0.73 PS + Ven) and the time spent in them (*p* < 0.05; 1.33 ± 1.46 PS vs. 49.25 ± 9.72 PS + Tia; 1.33 ± 1.46 PS vs. 49.80 ± 9.54 PS + Ven; Table 2 [38]).

### Biochemical Study

#### Chronic Administration of Antidepressant Drugs Normalized Changes, Evoked by Maternal Stress, in Levels of the Proinflammatory Cytokines in the Hippocampus and the Frontal Cortex of Adult Offspring Rats

Data have demonstrated the influence of various stressful events during the prenatal period on the immunological status of the brains of the offspring [40, 41]. In fact, we observed that the prenatal stress procedure upregulated microglia activation [42] as well as the expression of some proinflammatory cytokines in the brains of adult offspring [32]. Therefore, in the first set of experiments of the present paper, we evaluated the effect of chronic treatment with antidepressants on the changes, evoked by maternal stress, in the levels of the proinflammatory cytokines IL-1β and IL-18 in the hippocampus and frontal cortex of adult male rats.

**Fig. 2:**
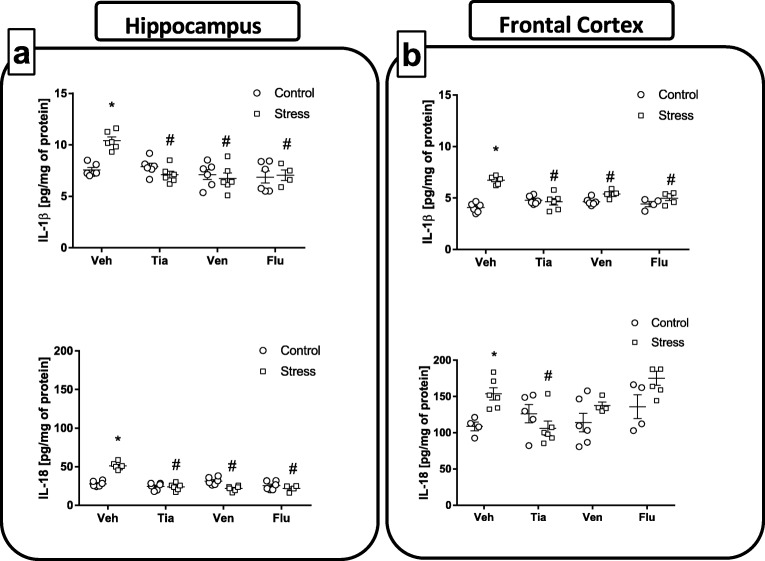
The effect of prenatal stress and antidepressant drugs treatment (tianeptine—Tia, venlafaxine—Ven, or fluoxetine—Flu) on the levels of pro-inflammatory (interleukin (IL)-1β, IL-18) and anti-inflammatory (IL-4) factors in the hippocampus (**a**) and frontal cortex (**b**). The data are presented as the means ± SEMs (pg/mg of protein), with *n* = 5–6 for each group. *p < 0.05 vs. control Veh group; #*p* < 0.05 vs. prenatally stressed Veh group. ANOVA (two-way), followed by Duncan’s test

*The hippocampus:* As shown in Fig. 2a, the results of ANOVA showed a significant increase in IL-1β (*F*_1,38_ = 3.11; 7.56 ± 0.24 Con vs. 10.41 ± 0.35 PS; *p* < 0.05) and IL-18 (*F*_1,38_ = 3.38; 27.86 ± 1.4 Con vs. 51.21 ± 1.84 PS; *p* < 0.05) levels in the hippocampus of prenatally stressed offspring compared to these levels in control offspring. Moreover, post hoc comparison revealed that chronic administration of all the antidepressant drugs (tianeptine, *p* < 0.05; venlafaxine, *p* < 0.05; fluoxetine, *p* < 0.05) normalized the increase, evoked by prenatal stress, in both proinflammatory cytokines (IL-1β—10.41 ± 0.35 PS vs. 7.10 ± 0.32 PS + Tia; 10.41 ± 0.35 PS vs. 6.73 ± 0.53 PS + Ven; 10.41 ± 0.35 PS vs. 7.05 ± 0.41 PS + Flu; IL-18—51.21 ± 1.84 PS vs. 23.85 ± 1.86 PS + Tia; 51.21 ± 1.84 PS vs. 21.87 ± 1.36 PS + Ven; 51.21 ± 1.84 PS vs. 21.91 ± 1.69 PS + Flu).

*The frontal cortex:* The examination of IL-1 β and IL-18 levels in the frontal cortex revealed that, in comparison to the control animals, prenatally stressed animals displayed increased IL-1β (*F*_1,36_ = 42.19; 4.06 ± 0.18 Con vs. 6.73 ± 0.15 PS; *p* < 0.05) and IL-18 (*F*_1,33_ = 7.86; 108.70 ± 4.89 Con vs. 153.5 ± 8.26 PS; *p* < 0.05) concentrations (Fig. 2b). Further post hoc examinations demonstrated that chronic administration of tianeptine (*p* < 0.05; 6.73 ± 0.15 PS vs. 4.63 ± 0.31 PS + Tia), venlafaxine (*p* < 0.05; 6.73 ± 0.15 PS vs. 5.39 ± 0.14 PS + Ven), and fluoxetine (*p* < 0.05; 6.73 ± 0.15 PS vs. 4.98 ± 0.21 PS + Flu) normalized the changes in IL-1β levels caused by prenatal stress. Interestingly, enhanced IL-18 levels produced by prenatal stress was only affected by chronic tianeptine administration (*p* < 0.05; 153.5 ± 8.26 PS vs. 106.26 ± 9.95 PS + Tia) (Fig. 2b).

#### Chronic Administration of Antidepressant Drugs Normalized Changes, Evoked by Maternal Stress, in the Protein Levels of Chemokine CCL2 and its Receptor CCR2 in the Hippocampus and the Frontal Cortex of Adult Offspring Rats

Data demonstrated that the chemokine CCL2 and its receptor CCR2 play a crucial role in the attraction of monocytes and other cells involved in the development of inflammatory responses [43]. Furthermore, numerous studies have focused on the suppression of CCL2–CCR2 axis as a way to reduce the damage characteristic of different brain disorders where the immune response is activated. Therefore, we also examined the impact of the antidepressants on protein expression in the CCL2–CCR2 axis in both the hippocampus and frontal cortex.

**Fig. 3:**
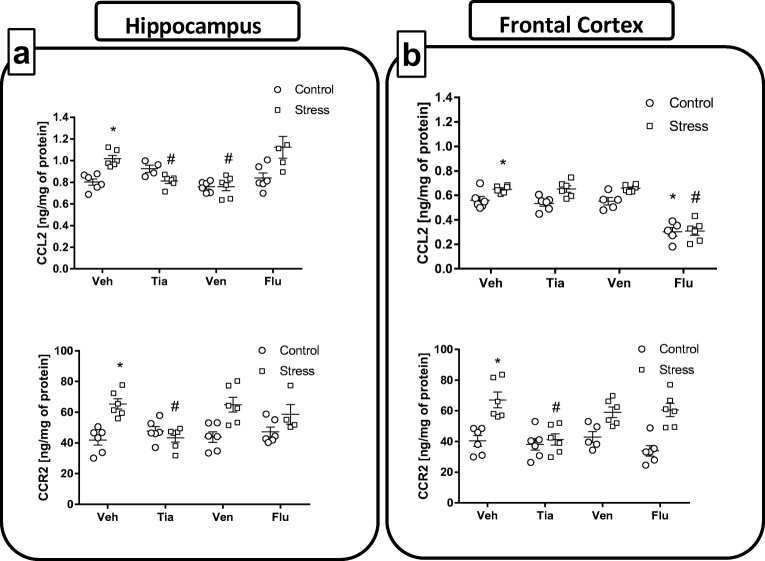
The effect of prenatal stress and antidepressant drugs treatment (tianeptine—Tia, venlafaxine—Ven, or fluoxetine—Flu) on the levels of CCL2 (ng/mg of protein) and its receptor—CCR2 (pg/mg of protein) in the hippocampus (**a**) and frontal cortex (**b**). The data are presented as the means ± SEMs, with *n* = 5–6 for each group. *p < 0.05 vs. control Veh group; #*p* < 0.05 vs. prenatally stressed Veh group. ANOVA (two-way), followed by Duncan’s test

*The hippocampus*: In the adult offspring of stressed females, we found a significant increase in levels of CCL2 (*F*_1,49_ = 18.49; 0.80 ± 0.03 Con vs. 1.01 ± 0.03 PS; *p* < 0.05; Fig. 3a) and its receptor CCR2 (*F*_1,48_ = 12.58; 41.87 ± 3.29 Con vs. 65.39 ± 3.37 PS; *p* < 0.05; Fig. 3a), compared to these levels in control offspring. Chronic administration of tianeptine (*p* < 0.05; 1.01 ± 0.03 PS vs. 0.81 ± 0.02 PS + Tia) and venlafaxine (*p* < 0.05; 1.01 ± 0.03 PS vs. 0.75 ± 0.03 PS + Ven) normalized the changes in CCL2 levels in the hippocampus caused by prenatal stress. Moreover, tianeptine (*p* < 0.05; 65.39 ± 3.37 PS vs. 43.34 ± 2.82 PS + Tia) administration was able also to normalize changes in CCR2 level evoked by stress (Fig. 3a).

*The frontal cortex:* In line with data obtained in hippocampus, analyses of the homogenized cortical samples also revealed a significant increase in CCL2 (*F*_1,48_ = 21.37; 0.56 ± 0.02 Con vs. 0.64 ± 0.01 PS; *p* < 0.05; Fig. 3b) and CCR2 (*F*_1,49_ = 42.10; 40.34 ± 3.49 Con vs. 67.14 ± 5.12 PS; *p* < 0.05; Fig. 3b) expression in prenatally stressed rats relative to expression levels in control rats. Chronic administration of tianeptine (*p* < 0.05; 67.14 ± 5.12 PS vs. 41.17 ± 3.69 PS + Tia) only normalized increases in CCR2 concentration evoked by prenatal stress (Fig. 3b). On the other hand, chronic treatment of fluoxetine statistically significantly diminished CCL2 levels in both controls (*p* < 0.05; 0.56 ± 0.02 Con vs. 0.30 ± 0.03 Con + Flu) and prenatally stressed adult offspring (*p* < 0.05; 0.64 ± 0.01 PS vs. 0.30 ± 0.03 PS + Flu).

#### Chronic Administration of Antidepressant Drugs Affected the iNOS Protein Levels, Evoked by Maternal Stress, in the Hippocampus and the Frontal Cortex of Adult Offspring Rats

Since the expression of iNOS is induced by certain proinflammatory stimuli, such as IL-1β, in the next set of experiments, we assessed the impact of chronic administration of antidepressants on iNOS levels in both hippocampal and frontal cortical homogenates of prenatally stressed rats using an ELISA assay.

**Fig. 4:**
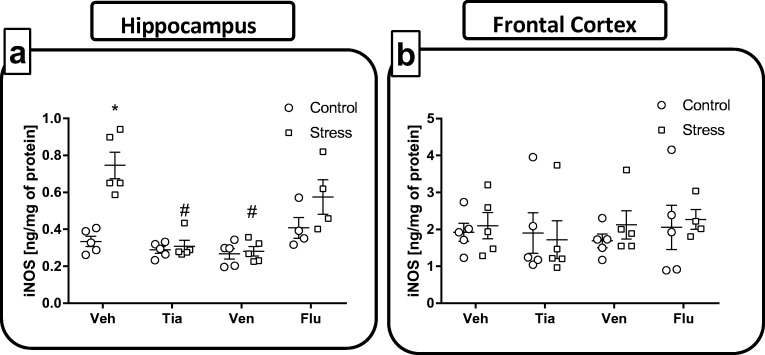
The effect of prenatal stress and antidepressant drugs treatment (tianeptine—Tia, venlafaxine—Ven, or fluoxetine—Flu) on the level of iNOS (ng/mg of protein) in the hippocampus (**a**) and frontal cortex (**b**). The data are presented as the means ± SEMs, with *n* = 5–6 for each group. *p < 0.05 vs. control Veh group; #*p* < 0.05 vs. prenatally stressed Veh group. ANOVA (two-way), followed by Duncan’s test

*The hippocampus*: Our data demonstrated that the prenatal stress procedure significantly increased iNOS level (*F*_1,43_ = 2.11; 0.33 ± 0.02 Con vs. 0.74 ± 0.07 PS; *p* < 0.05, Fig. 4a), compared to the control procedure. ANOVA revealed that chronic administration of tianeptine (*p* < 0.05; 0.74 ± 0.07 PS vs. 0.30 ± 0.03 PS + Tia) and venlafaxine (*p* < 0.05; 0.74 ± 0.07 PS vs. 0.28 ± 0.02 PS + Ven) attenuated the upregulation in iNOS protein concentration evoked by stress (Fig. 4a).

*The frontal cortex:* In contrast, there was no impact of either prenatal stress or treatment with antidepressant drugs on the iNOS levels in frontal cortex of adult offspring rats (Fig. 4b). These observations clearly demonstrate the brain structure-dependent impact of prenatal stress and antidepressant administration on iNOS levels.

#### The Impact of Chronic Administration of Antidepressant Drugs on the TLR4-Related Pathways in the Hippocampus and the Frontal Cortex of Adult Offspring Rats Exposed to Prenatal Stress

TLR4 is known to be one of the major inflammatory signaling receptors that leads to activation of the MyD88 adapter protein and transcription factors, including NFκB, which consequently leads to synthesis of inflammatory genes. Thus, we examined the influence of prenatal stress and chronic treatment with antidepressant drugs on the TLR4 receptor and MyD88 adapter protein levels.

**Fig. 5:**
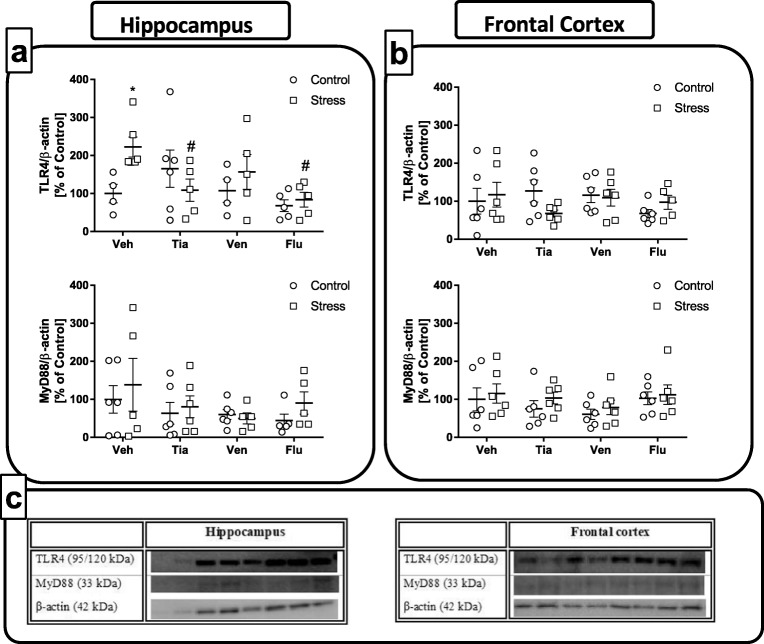
The effect of prenatal stress and antidepressant drugs treatment (tianeptine—Tia, venlafaxine—Ven, or fluoxetine—Flu) on the levels of TLR4 and its adapter protein—MyD88 in the hippocampus (**a**) and frontal cortex (**b**). (**c**) Representative immunoblots. The bands from left: 1, control; 2, stress; 3, control + TIA; 4, stress + TIA; 5, control + VEN; 6, stress + VEN; 7, control + FLU; 8, stress + FLU. The data are presented as the means ± SEMs, with *n* = 5–6 for each group. *p < 0.05 vs. control Veh group; #*p* < 0.05 vs. prenatally stressed Veh group. ANOVA (two-way), followed by Duncan’s test

*The hippocampus*: As shown in Fig. 5, prenatal stress enhanced TLR4 levels (*F*_1,32_ = 1.84; 100 ± 19.97 Con vs. 226.64 ± 26.32 PS; *p* < 0.05, panel a). Among the chronically administered antidepressants, ANOVA revealed a significant impact of tianeptine (*p* < 0.05; 226.64 ± 26.32 PS vs. 108.75 ± 26.74 PS + Tia) and fluoxetine (*p* < 0.05; 226.64 ± 26.32 PS vs. 83.91 ± 17.84 PS + Flu) on the changes evoked by maternal stress. In the case of venlafaxine, we only observed a tendency to diminish this parameter (*p* < ns). Although we found that the prenatal stress procedure stimulated MyD88 expression, this change did not reach statistical significance (Fig. 5a). Similarly, although we observed the normalizing tendency of all chronically administered drugs on MyD88 protein levels induced by stress, this effect was not statistically significant.

*The frontal cortex:* In contrast to the hippocampus, we did not observe an impact of prenatal stress nor all applied antidepressants on the TLR4 levels (Fig. 5b). Moreover, we did not observe statistically significant changes in MyD88 expression after the prenatal stress procedure or chronic treatment with the antidepressant drugs (Fig. 5b). Thus, our results highlighted the brain structure-dependent impact of antidepressants on the changes in TLR4 levels evoked by maternal stress.

#### The Impact of Chronic Administration of Antidepressant Drugs on the NFкB Signaling Pathway in the Hippocampus and the Frontal Cortex of Adult Offspring Rats Exposed to Prenatal Stress

In the next set of experiments, we examined the effect of chronic antidepressant drug administration on the phosphorylation level of the p65 NFκB subunit and IκB protein, an inhibitor of the NFκB complex, in the hippocampus and the frontal cortex of prenatally stressed offspring.

**Fig. 6:**
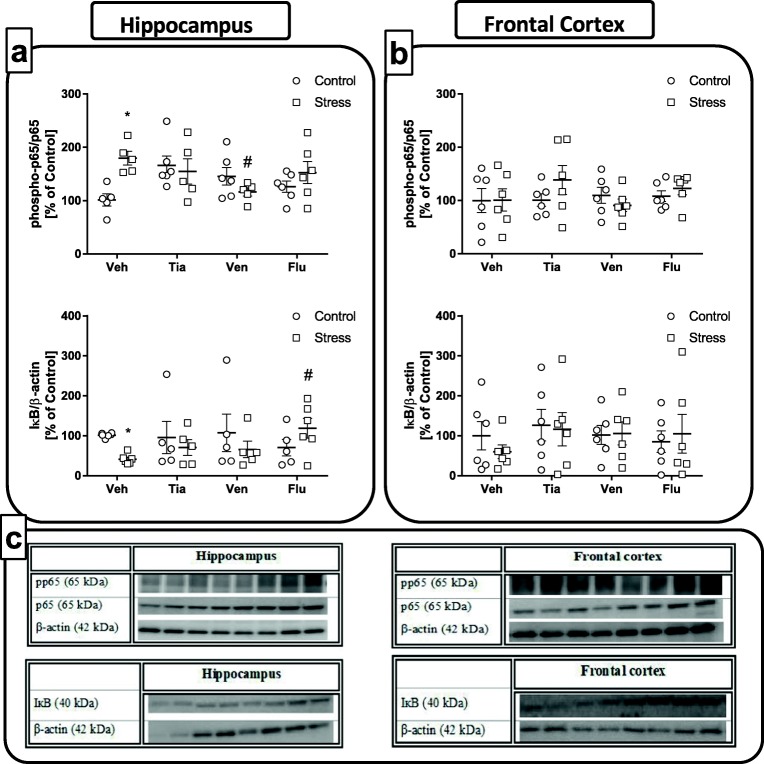
The effect of prenatal stress and antidepressant drugs treatment (tianeptine—Tia, venlafaxine—Ven, or fluoxetine—Flu) on the phosphorylation level of the p65 NFκB subunit and IκB protein, an inhibitor of the NFκB complex, in the hippocampus (**a**) and frontal cortex (**b**). (**c**) Representative immunoblots. The bands from left: 1, control; 2, stress; 3, control + TIA; 4, Stress + TIA; 5, control + VEN; 6, stress + VEN; 7, control + FLU; 8, stress + FLU. The data are presented as the means ± SEMs, with *n* = 5–6 for each group. *p < 0.05 vs. control Veh group; #*p* < 0.05 vs. prenatally stressed Veh group. ANOVA (two-way), followed by Duncan’s test

*The hippocampus:* As shown in Fig. 6, ANOVA showed a significant increase in the phosphorylation of the p65 subunit in the hippocampus (*F*_1,37_ = 3.20; 100 ± 12.10 Con vs. 179.62 ± 12.69 PS; *p* < 0.05, panel a) of prenatally stressed offspring compared to phosphorylation levels in the control offspring. Among the tested antidepressants, post hoc comparisons found that venlafaxine (*p* < 0.05; 179.62 ± 12.69 PS vs. 126.41 ± 12.04 PS + Ven) normalized this effect. Interestingly, we observed diminished protein levels of IкB (*F*_1,35_ = 4.41; 100 ± 2.55 Con vs. 45.46 ± 4.33 PS; *p* < 0.05) in rats after the prenatal stress procedure, and among the antidepressants, only fluoxetine treatment was able to normalize the changes evoked by stress (*p* < 0.05; 45.46 ± 4.33 PS vs. 118.90 ± 24.64 PS + Flu).

*The frontal cortex:* Analyses of samples obtained from the frontal cortex of adult rats subjected to a prenatal stress procedure showed that neither prenatal stress nor antidepressant drug administration affected the phosphorylation level of the p65 NFκB subunit (*F*_1,40_ = 0.49) or IκB protein levels (*F*_1,39_ = 0.09; Fig. 6b). Our data demonstrated that chronic treatment with antidepressants only slightly affected the NFкB signaling pathway in the hippocampus of adult offspring.

#### The Impact of Chronic Administration of Antidepressant Drugs on the Levels of Protein in the NLRP3 Inflammasome Signaling Pathway in the Hippocampus and the Frontal Cortex of Adult Offspring Rats Exposed to Prenatal Stress

Recent data described the TLR4-mediated induction of the NLRP3 inflammasome. Moreover, it has been found that NFкB is a central mediator in the “priming signal” of NLRP3 inflammasome activation, which leads to stimulation of the enzyme caspase 1, which is responsible for the generation of the mature form of proinflammatory cytokines, mostly IL-1β and IL-18. Therefore, in the last set of experiments, we focused on the effect of chronic administration of antidepressant drugs on the protein levels of all the NLRP3 inflammasome subunits, i.e., NLRP3, caspase-1, and ASC, in prenatally stressed offspring.

**Fig. 7:**
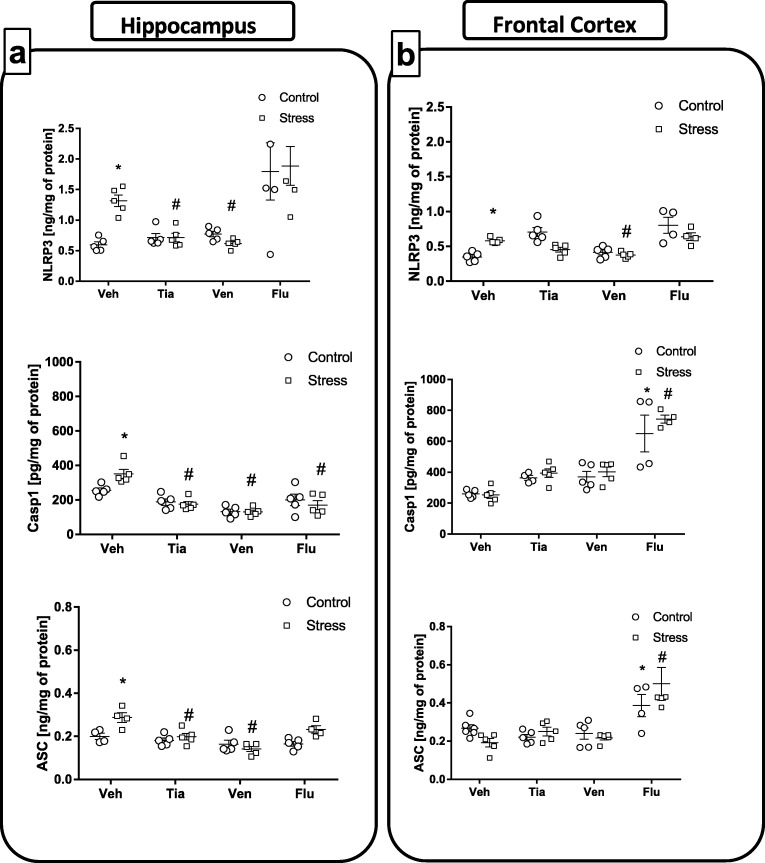
The effect of prenatal stress and antidepressant drugs treatment (tianeptine—Tia, venlafaxine—Ven, or fluoxetine—Flu) on the protein levels of all the NLRP3 inflammasome subunits, i.e., NLRP3 (ng/mg of protein), caspase-1 (pg/mg of protein), and ASC (ng/mg of protein) in the hippocampus (**a**) and frontal cortex (**b**). The data are presented as the means ± SEMs, with *n* = 5–6 for each group. *p < 0.05 vs. control Veh group; #*p* < 0.05 vs. prenatally stressed Veh group. ANOVA (two-way), followed by Duncan’s test

*The hippocampus*: Regarding the differences between control and prenatally stressed rats, a significant increase in NLRP3 (*F*_1,32_ = 1.25; 0.59 ± 0.04 Con vs. 1.11 ± 0.08 PS; *p* < 0.05), Casp-1 (*F*_1,32_ = 0.73; 255.74 ± 12.34 Con vs. 351.30 ± 24.44 PS; *p* < 0.05) and ASC (*F*_1,25_ = 9.72; 0.19 ± 0.01 Con vs. 0.28 ± 0.01 PS; *p* < 0.05) subunit protein levels was observed (Fig. 7a). Among the tested antidepressants, post hoc comparisons found that tianeptine (*p* < 0.05; NLRP3—1.11 ± 0.08 PS vs. 0.71 ± 0.06 PS + Tia; ASC—0.28 ± 0.01 PS vs. 0.19 ± 0.01 PS + Tia) and venlafaxine (*p* < 0.05; NLRP3—1.11 ± 0.08 PS vs. 0.61 ± 0.03 PS + Ven; ASC—0.28 ± 0.01 PS vs. 0.14 ± 0.01 PS + Ven) treatments normalized changes in levels of NLRP3 and ASC subunits in prenatally stressed offspring (Fig. 7a). Importantly, all antidepressant drugs tested, i.e., tianeptine (*p* < 0.05; 351.30 ± 24.44 PS vs. 175.23 ± 14.09 PS + Tia), venlafaxine (*p* < 0.05; 351.30 ± 24.44 PS vs. 130.62 ± 9.99 PS + Ven), and fluoxetine (*p* < 0.05; 351.30 ± 24.44 PS vs. 169.61 ± 24.07 PS + Flu), significantly attenuated the increase in the level of Casp-1 subunit (Fig. 7a) induced by the prenatal stress procedure.

*The frontal cortex*: Our experiments showed that the prenatal stress procedure significantly increased the NLRP3 subunit (*F*_1,30_ = 2.40; 0.34 ± 0.02 Con vs. 0.58 ± 0.01 PS; *p* < 0.05; Fig. 7b) levels in the frontal cortex. Post hoc comparisons showed that only chronic administration of venlafaxine (*p* < 0.05; 0.58 ± 0.01 PS vs. 0.37 ± 0.01 PS + Ven) normalized changes evoked by prenatal stress in the frontal cortex. No significant changes in the Casp-1 (*F*_1,43_ = 6.37) and ASC (*F*_1,56_ = 0.99) subunit levels were found in prenatally stressed animals in comparison to controls. Moreover, we showed that fluoxetine (*p* < 0.05) surprisingly upregulated the levels of both caspase-1 and ASC subunits in control (caspase-1—259.60 ± 10.40 Con vs. 650.75 ± 97.02 Con + Flu; ASC—0.24 ± 0.01 Con vs. 0.38 ± 0.04 Con + Flu) and prenatally stressed offspring (caspase-1—253.66 ± 20.09 PS vs. 743.90 ± 21.04 PS + Flu; ASC—0.17 ± 0.02 PS vs. 0.50 ± 0.07 PS + Flu; Fig. 7b).

## Discussion

The most important finding presented in our study is that chronically administered antidepressant drugs attenuated changes in inflammatory status evoked by prenatal stress procedure in brain areas in adult offspring rats, including IL-1β and IL-18 expression, iNOS inhibition, and CCL2–CCR2 axis modulation and accompanied by an improvement in behavioral dysfunctions. Moreover, our data provide evidence that the beneficial, anti-inflammatory effect of antidepressants, particularly in the hippocampus, points to the inhibition of NLRP3 inflammasome-activated pathways as a possible mechanism of action for these drugs.

Data indicate that early adverse experiences may play a crucial role in the pathogenesis of depression through malfunction of the brain immune system regulation [10]. Prenatal stress procedures, which are commonly accepted animal models of depression [30, 32, 42, 44–48], differs from other stress-related models of depression because in the animals exposed to stressful conditions in the prenatal phase, the behavioral, neurochemical, and immunological changes induced during neurodevelopment are long lasting [47–50]. In the present study, we confirmed the behavioral disturbances in the offspring of rat dams that were stressed during the last week of pregnancy, expressed as an increase in immobility time and a decrease in swimming and climbing behavior in the modified Porsolt swim test. Furthermore, increased anxiety-like behavior expressed as a reduction in the number of entries into the open arms of the maze and a decrease in the time spent in them was observed. Moreover, we pharmacologically validated this model showing that chronic treatment with various antidepressant drugs, i.e., tianeptine, venlafaxine, or fluoxetine, normalized behavioral disturbances evoked by the stress, a finding that could be interpreted as an attenuation of depressive-like and anxiety-like behaviors. Since previously published data demonstrated that in rats, prenatal stress profoundly affected the offspring’s behavior via immune alterations [5, 51], the main purpose of present paper was to characterize the molecular impact of treatment with various antidepressants on the changes in proinflammatory factors levels in the hippocampus and frontal cortex of adult offspring prenatally exposed to stress.

The present study demonstrated that the stress-induced release of IL-1β in hippocampus and frontal cortex were normalized by tianeptine, venlafaxine, and fluoxetine chronic administration. Furthermore, the IL-18 levels elevated by stress in the hippocampus were diminished by tianeptine, venlafaxine, and fluoxetine, however, only by tianeptine administration in frontal cortex. The beneficial anti-inflammatory properties of tianeptine and venlafaxine in hippocampus were confirmed by the ability of those drugs to decrease iNOS expression that was upregulated by prenatal stress.

Many studies have highlighted the significance of IL-1β as a pivotal mediator of stress-related disorders including depression [52–55]. Among them, experimental data using a restraint stress model in mice demonstrated a higher expression of interleukin 1β (IL-1β) in the hippocampus [56]. In a chronic mild stress (CMS) model of depression, higher concentrations of IL-1β and IL-6 in the brain and IL-6 and TNF-α in serum were shown [57]. A majority of the clinical data reported increases in IL-1β levels in depressed patients in the periphery [58, 59] and identified a role of this cytokine in response to treatment with antidepressants [54], as well as a possible marker of depression [60]. In fact, 30% of depressed patients, who are resistant to selective serotonin reuptake inhibitor therapy, have significantly higher IL-1β and/or IL-18 serum levels [61, 62]. However, constitutive levels of the IL-1 family of proinflammatory cytokines in the brain are required for physiological brain functioning, including the mechanisms of learning, memory, cognition [55], neuronal genesis, and survival, as well as HPA axis sensitivity regulation [63]. In contrast, prolonged high levels of IL-1β in the brain has been identified as the first step in a harmful cascade of other proinflammatory factors, including activation of the chemoattractant chemokine CCL2 acting through its main brain receptor CCR2 as well as iNOS production [26]. This cascade affects neurogenesis along with a reduction in the size of hippocampus, as well as serotonergic metabolism, and may be a cause of depression [64]. Interestingly, data have suggested that CCL2–CCR2 axis and IL-1β, as well as IL-18, may be regulated through a feedback mechanism in the brain [11, 65]. Since a strong link between inflammatory cytokines and CCL2–CCR2 axis has been suggested, the question arises whether chronic treatment with antidepressants may modulate changes in the CCL2–CCR2 axis evoked by prenatal stress.

Data from our study demonstrated that the stress-induced increase in the CCL2 levels were normalized in the hippocampus by chronic tianeptine and venlafaxine administration. Moreover, the enhanced CCR2 levels in both brain areas of prenatally stressed rats was affected by tianeptine treatment. So far, data concerning CCL2–CCR2 axis regulation in animal model of depression are contradictory. We previously reported that in young prenatally stressed offspring, the hippocampal levels of CCL2 were upregulated [42]. Moreover, in microglia cultures (obtained from 1- to 2-day-old pups), we observed the harmful impact of the stress procedure on the CCL2–CCR2 expression levels. Therefore, based on our data, it may be suggested that changes in the prenatal environment may contribute to the onset of long-lasting malfunction in the CCL2–CCR2 axis [42]; however, the importance of this protein system as a target in the pharmacotherapy still remains controversial. This is because during inflammation, the CCL2–CCR2 axis acts in concert with selectins and integrins to cause the attraction of monocytes and other cells to the site of inflammation [65, 66], and the potency of CCL2 as an important neuromodulator has been recently documented [67]. In addition, studies have postulated implications of the CCL2–CCR2 axis in neuronal communication and even neuronal regeneration [68, 69]. Moreover, there are data showing that CCL2 treatment of microglia led to the increase in migration and proliferation of these cells and regulation of its proinflammatory phenotype [70, 71]. While the concept of an increase in the concentration of proinflammatory cytokines within the brain during stress-related depression is now established, the most gripping objective in our study was to determine the potential mechanism of action of these antidepressant drugs on the inflammatory status evoked by the prenatal stress procedure in adult male rats.

The NLRP3 inflammasome activation links cytokines, psychological stress, and depression [26, 72, 73]. For example, evidence indicates that the NLRP3 inflammasome platform contributes to IL-1β and IL-18 release [55, 74, 75]. It has been found that the NLRP3 inflammasome requires a double signal for activation. First, the priming signal facilitated through the activation of TLR4 on the cell surface by stress, LPS administration or other factors [14, 23, 62, 76] leads to the formation of the TLR4/myeloid differentiation protein 2 (MD-2) complex and subsequent recruitment of an intracellular adaptor protein, MyD88, which then activates transcription factor NFкB and NLRP3-dependent formation of inactive forms of cytokines (proIL-1β and proIL-18). Therefore, we evaluated first the effect of antidepressants on NFкB as a transcriptional activator of the NLRP3 inflammasome [73, 77] in prenatally stressed rats. We reported, for the first time, that chronic tianeptine and fluoxetine administration attenuated upregulation of hippocampal TLR4 protein expression evoked by prenatal stress. Moreover, in our study, we found a beneficial impact of antidepressants on p65 and IκB subunits of the NFкB complex. In fact, chronic treatment of venlafaxine suppressed the stress-induced phosphorylation of serine, which is important in initiating transcription of the p65 NFкB subunit, while fluoxetine normalized downregulation of IκB degradation in hippocampus that was evoked by the prenatal stress procedure. Taking into account studies that reported participation of the NFкB pathway in IL-1β-stimulated CCL2 protein release [65], which in our study was normalized tianeptine and venlafaxine administration, we can postulate the complex transcriptional regulation of NLRP3 inflammasome activation by chronic antidepressant treatment preferentially in the hippocampus of prenatally stressed offspring. The posttranscriptional NLRP3 inflammasome regulation led to activation of NLRP3 inflammasome components, including inactive NLRP3, proIL-1β, and proIL-18, and with the participation of ASC protein, to the formation of the active form of caspase-1. The activated caspase-1 is indispensable for the generation of active forms of IL-1β and IL-18 [78].

The most intriguing finding in our paper was the observation that tianeptine and venlafaxine chronic treatment normalized in the hippocampus, the overactivation evoked by prenatal stress of all NLRP3 inflammasome subunits, i.e., NLRP3, ASC, and caspase-1 levels, while fluoxetine only normalized caspase-1. On the other hand, stress-induced increases in the NLRP3 subunit level in the frontal cortex was attenuated only by venlafaxine administration. The divergent potency of antidepressants in the brain areas under study in the regulation of NLRP3 inflammasome activation may be partially explained by the fact that the hippocampus is a structure particularly sensitive to stressful stimuli and, in consequence, to neuroimmune modulation [5, 79], and may suggest the engagement of other pathways being responsible for the anti-inflammatory properties of antidepressant drugs in the frontal cortex in our model of depression. Since Pan et al. [73] demonstrated the involvement of the NLRP3 inflammasome pathway in the anti-inflammatory action of fluoxetine in the frontal cortex in a chronic mild stress model of depression, we can also postulate that the differences may be attributed also to the experimental procedure, animal strain, or detection methods used.

Thus far, data regarding the impact of antidepressants on the NLRP3 inflammasome are scarce. However, the association between the effects of fluoxetine on the NLRP3 complex in the hippocampus has been evaluated [73]. Moreover, the suppressive effects of fluoxetine on the chronic mild stress-induced NLRP3 inflammasome activation in the hippocampus and in the periphery via downregulated ROS–PKR–NLRP3 signaling pathways in macrophages and microglia has been demonstrated [80]. In addition, in the hippocampus and frontal cortex of animals subjected to the chronic unpredictable mild stress procedure, researchers showed higher levels of IL-1β, NLRP3, its subunits, and TLR2, and what is more, fluoxetine normalized these effects [81, 82]. Recently, we demonstrated that LPS evoked an upregulation in NLRP3 inflammasome activation in primary microglia that was attenuated by tianeptine pretreatment [83]. On the other hand, only a few clinical reports have shown that caspase-1, NLRP3 mRNA expression, and NLRP3 protein levels are increased in the peripheral blood mononuclear cells [14], and these effects were reversed by tricyclic antidepressant amitriptyline treatment [62]. Therefore, based on mentioned above data, we can postulate that our results are the first to provide the NLRP3 inflammasome in the hippocampus as a new, sensitive pharmacological target for antidepressant drugs with various mechanisms of action, i.e., tianeptine, venlafaxine, and fluoxetine, and suggest an interesting therapeutic strategy for the modulation and treatment of depression, which may be accompanied by improvements in the behavioral dysfunctions evoked by prenatal stress. On the other hand, considering very diverse effects of antidepressant drugs on other protein systems and their interrelationships, it is difficult to draw unequivocal mechanistic interpretation about the one basis underlying antidepressant drug action in the prenatal stress model, which is some limitation of our study.

In conclusion, it is clear that the discovery of the role of NLRP3 inflammasome activation in the mechanisms of antidepressant action has opened an array of research opportunities to investigate inflammasome-targeted therapies for depression and other pathological changes in the brain; however, further study in larger populations examining the impact of these antidepressants on the assembly of the NLRP3 inflammasome is urgently needed.

## Electronic Supplementary Material

ESM 1 (PPT 3288 kb)
