# Supplementary material for: Role of Chronic Administration of Antidepressant Drugs in the Prenatal Stress-Evoked Inflammatory Response in the Brain of Adult Offspring Rats: Involvement of the NLRP3 Inflammasome-Related Pathway
Source: Mol Neurobiol. 2019 Jan 4;56(8):5365–80. doi: 10.1007/s12035-018-1458-1 (PMC6614144; doi:10.1007/s12035-018-1458-1)
Supplement: Supplementary file 1 — (PPT 3288 kb) [file 12035_2018_1458_MOESM1_ESM.ppt]

## Slide 1
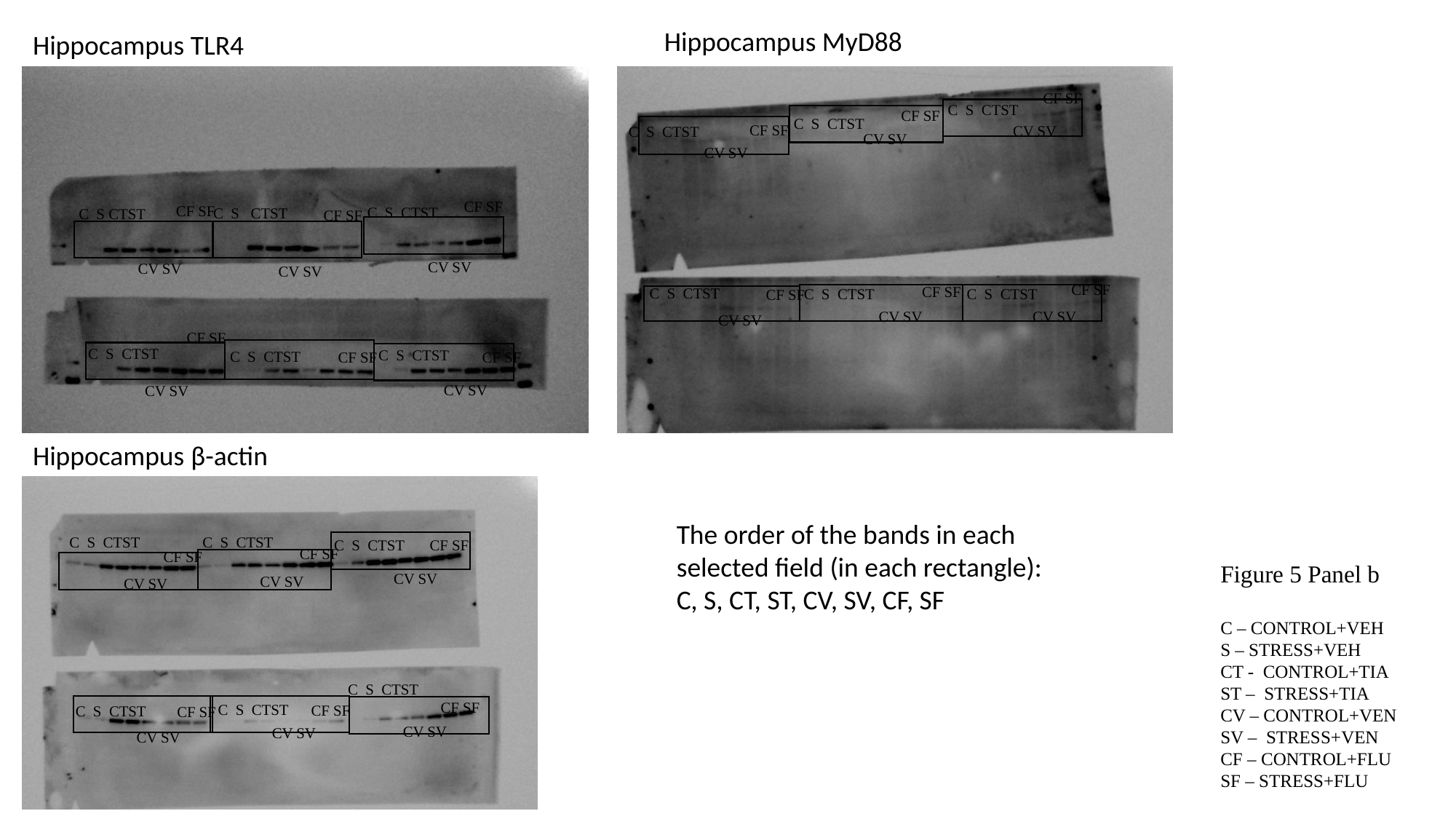

Hippocampus MyD88
Hippocampus TLR4
CF SF
C S CTST
CF SF
C S CTST
CF SF
CV SV
C S CTST
CV SV
CV SV
CF SF
CF SF
C S CTST
C S CTST
C S CTST
CF SF
CV SV
CV SV
CV SV
CF SF
CF SF
C S CTST
C S CTST
C S CTST
CF SF
CV SV
CV SV
CV SV
CF SF
C S CTST
C S CTST
C S CTST
CF SF
CF SF
CV SV
CV SV
Hippocampus β-actin
The order of the bands in each selected field (in each rectangle):
C, S, CT, ST, CV, SV, CF, SF
C S CTST
C S CTST
CF SF
C S CTST
CF SF
CF SF
Figure 5 Panel b
CV SV
CV SV
CV SV
C – CONTROL+VEH
S – STRESS+VEH
CT - CONTROL+TIA
ST – STRESS+TIA
CV – CONTROL+VEN
SV – STRESS+VEN
CF – CONTROL+FLU
SF – STRESS+FLU
C S CTST
CF SF
C S CTST
CF SF
C S CTST
CF SF
CV SV
CV SV
CV SV

## Slide 2
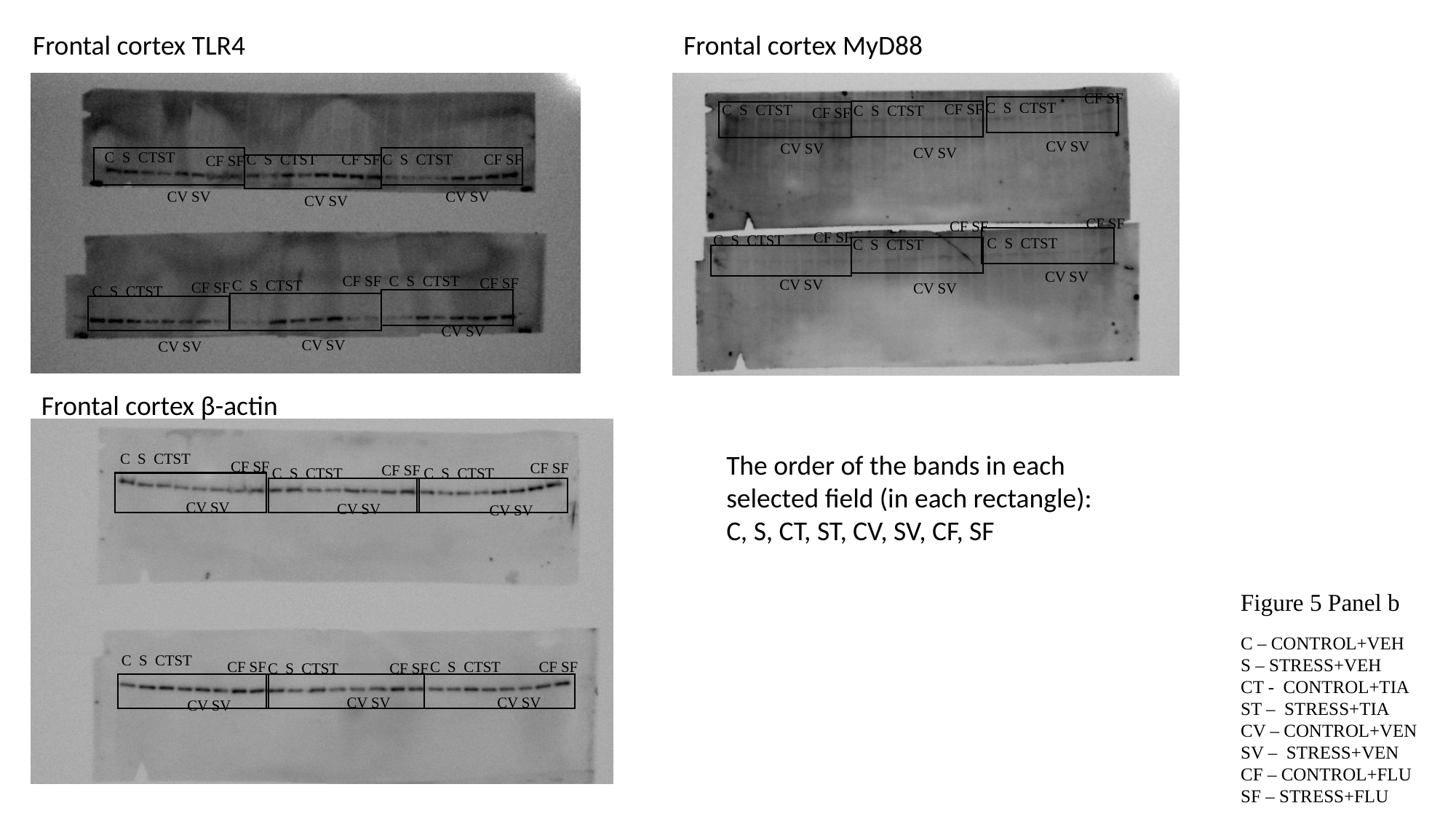

Frontal cortex TLR4
Frontal cortex MyD88
CF SF
C S CTST
CF SF
C S CTST
C S CTST
CF SF
CV SV
CV SV
CV SV
C S CTST
C S CTST
C S CTST
CF SF
CF SF
CF SF
CV SV
CV SV
CV SV
CF SF
CF SF
CF SF
C S CTST
C S CTST
C S CTST
CV SV
C S CTST
CF SF
CF SF
CV SV
C S CTST
CF SF
CV SV
C S CTST
CV SV
CV SV
CV SV
Frontal cortex β-actin
The order of the bands in each selected field (in each rectangle):
C, S, CT, ST, CV, SV, CF, SF
C S CTST
CF SF
CF SF
CF SF
C S CTST
C S CTST
CV SV
CV SV
CV SV
Figure 5 Panel b
C – CONTROL+VEH
S – STRESS+VEH
CT - CONTROL+TIA
ST – STRESS+TIA
CV – CONTROL+VEN
SV – STRESS+VEN
CF – CONTROL+FLU
SF – STRESS+FLU
C S CTST
C S CTST
CF SF
CF SF
C S CTST
CF SF
CV SV
CV SV
CV SV

## Slide 3
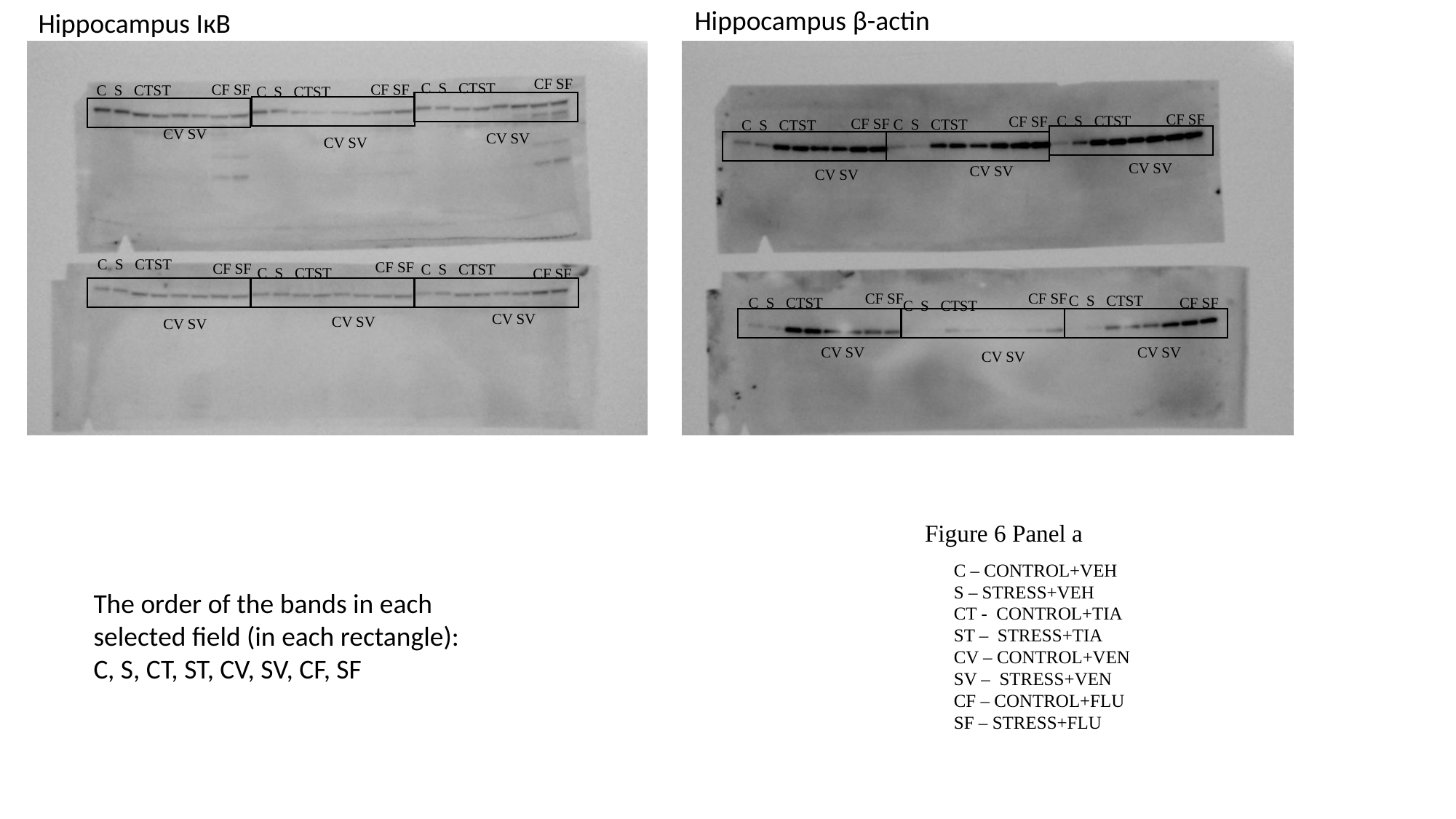

Hippocampus IкB
Hippocampus β-actin
CF SF
C S CTST
CF SF
CF SF
C S CTST
C S CTST
CF SF
C S CTST
CF SF
CF SF
C S CTST
C S CTST
CV SV
CV SV
CV SV
CV SV
CV SV
CV SV
C S CTST
CF SF
CF SF
C S CTST
C S CTST
CF SF
CF SF
CF SF
C S CTST
C S CTST
CF SF
C S CTST
CV SV
CV SV
CV SV
CV SV
CV SV
CV SV
Figure 6 Panel a
C – CONTROL+VEH
S – STRESS+VEH
CT - CONTROL+TIA
ST – STRESS+TIA
CV – CONTROL+VEN
SV – STRESS+VEN
CF – CONTROL+FLU
SF – STRESS+FLU
The order of the bands in each selected field (in each rectangle):
C, S, CT, ST, CV, SV, CF, SF

## Slide 4
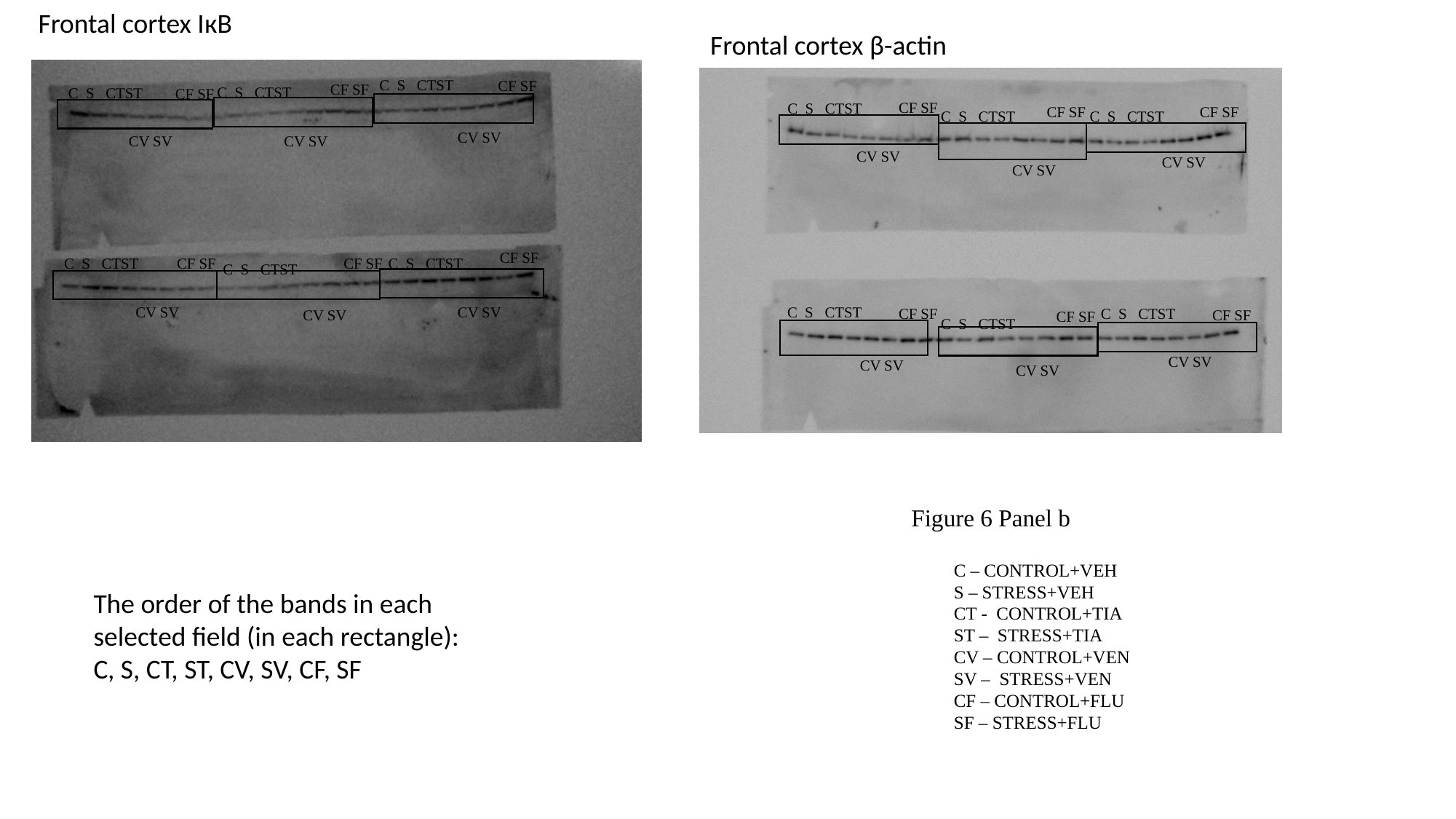

Frontal cortex IкB
Frontal cortex β-actin
C S CTST
CF SF
CF SF
C S CTST
C S CTST
CF SF
CF SF
C S CTST
CF SF
CF SF
C S CTST
C S CTST
CV SV
CV SV
CV SV
CV SV
CV SV
CV SV
CF SF
C S CTST
CF SF
CF SF
C S CTST
C S CTST
CV SV
CV SV
C S CTST
CF SF
C S CTST
CF SF
CV SV
CF SF
C S CTST
CV SV
CV SV
CV SV
Figure 6 Panel b
C – CONTROL+VEH
S – STRESS+VEH
CT - CONTROL+TIA
ST – STRESS+TIA
CV – CONTROL+VEN
SV – STRESS+VEN
CF – CONTROL+FLU
SF – STRESS+FLU
The order of the bands in each selected field (in each rectangle):
C, S, CT, ST, CV, SV, CF, SF

## Slide 5
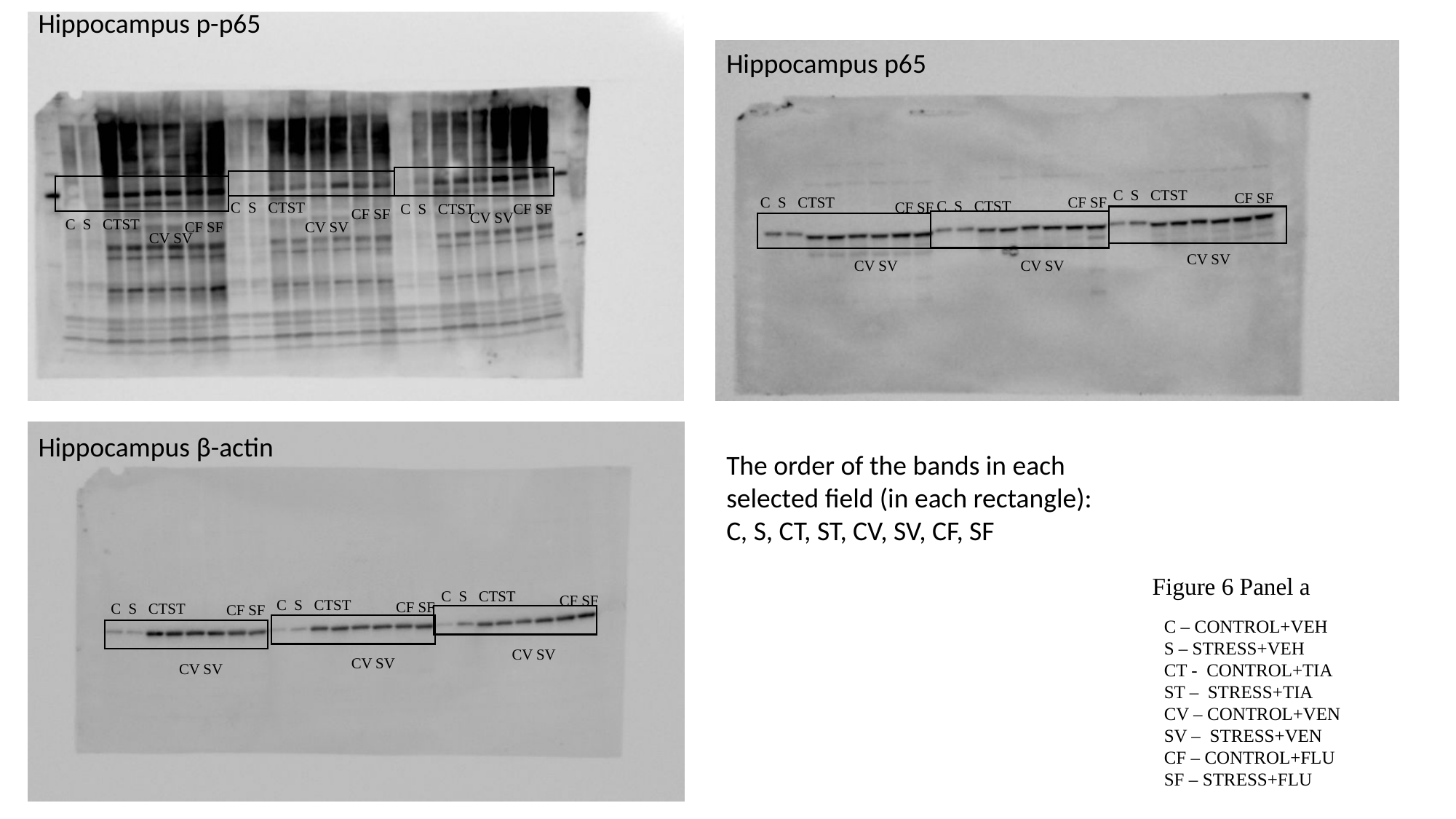

Hippocampus p-p65
Hippocampus p65
C S CTST
CF SF
C S CTST
CF SF
C S CTST
CF SF
C S CTST
C S CTST
CF SF
CF SF
CV SV
C S CTST
CV SV
CF SF
CV SV
CV SV
CV SV
CV SV
Hippocampus β-actin
The order of the bands in each selected field (in each rectangle):
C, S, CT, ST, CV, SV, CF, SF
Figure 6 Panel a
C S CTST
CF SF
C S CTST
CF SF
C S CTST
CF SF
C – CONTROL+VEH
S – STRESS+VEH
CT - CONTROL+TIA
ST – STRESS+TIA
CV – CONTROL+VEN
SV – STRESS+VEN
CF – CONTROL+FLU
SF – STRESS+FLU
CV SV
CV SV
CV SV

## Slide 6
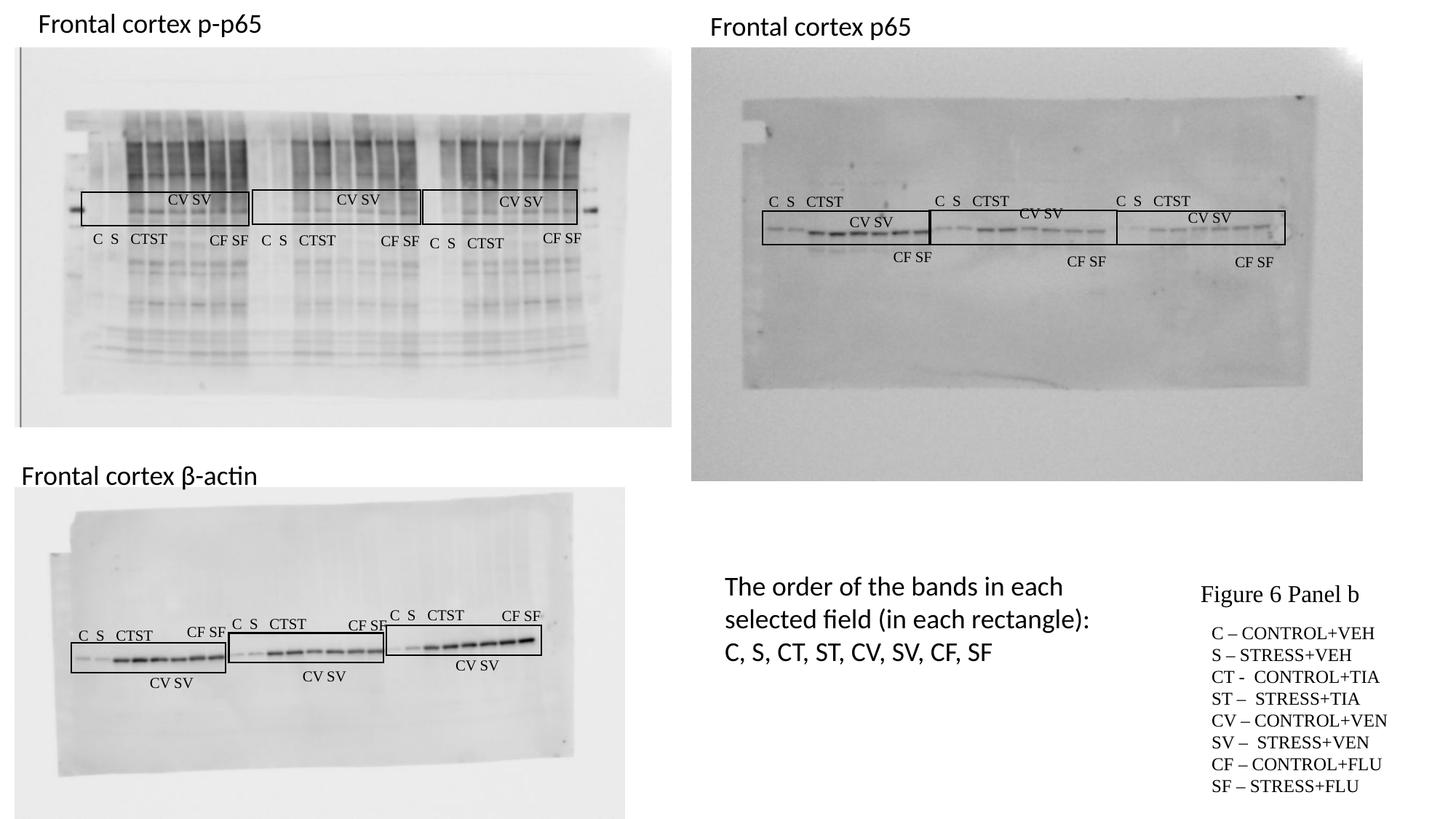

Frontal cortex p-p65
Frontal cortex p65
CV SV
CV SV
C S CTST
C S CTST
CV SV
C S CTST
CV SV
CV SV
CV SV
CF SF
C S CTST
CF SF
C S CTST
CF SF
C S CTST
CF SF
CF SF
CF SF
Frontal cortex β-actin
The order of the bands in each selected field (in each rectangle):
C, S, CT, ST, CV, SV, CF, SF
Figure 6 Panel b
C S CTST
CF SF
C S CTST
CF SF
C – CONTROL+VEH
S – STRESS+VEH
CT - CONTROL+TIA
ST – STRESS+TIA
CV – CONTROL+VEN
SV – STRESS+VEN
CF – CONTROL+FLU
SF – STRESS+FLU
CF SF
C S CTST
CV SV
CV SV
CV SV
